# Supplementary material for: Profile of pro-inflammatory cytokines in colostrum of nursing mothers at the extremes of reproductive age
Source: PLoS One. 2020 Jun 16;15(6):e0231882. doi: 10.1371/journal.pone.0231882 (PMC7297348; doi:10.1371/journal.pone.0231882)
Supplement: Attachment — Submitted filename: PLOS_rev_colos.docx [file pone.0231882.s001.docx]

Review:	Manuscript number: PONE-D-19-35580

General comments:

The manuscript describes the profile of cytokines in mothers at early (10-24 years) vis-à-vis those at late gestational age (≥35 years) relative to a control group (25-34).The study is of interest considering that both populations are within the high risk group for complications for mother and infant. Colostrum is notably for defense and protection of the baby, rather than as a nutrient so assessment of cytokine profiles is relevant.

The article can be improved with some help in the style of writing and in the grammar. Details on specific sections are presented below.

Title: The title could benefit from a minor modification: cytokines are either pro- or anti-inflammatory or both so “selected” could replace “inflammatory” in the title.

Keywords: I suggest you replace “extremes age” with “ age extremes”

Abstract:

Line 39: the age classification is incorrect in the abstract

Methods: The methods section of the abstract needs significant editing to improve the sentence structure and grammar. The results section also need to be improved.

Introduction:

Line 89: …glycoproteins that signal…….

Line 92: ….and function of the newborn’s immune……………

Line 105 : reference numbering has an error

Line 106-107: Please improve the sentence.

Methods

Lines 124-125: Please improve the sentence

Line 133-137: Please revise

Lines 138-139: The sentence is too long. Break it up appropriately.

Lines 142-147: Please revise

Lines 151-152: The dependent variables are not determination of the concentrations……..

Lines 154-155: Please write out the full meaning of the abbreviated letters

Lines 159 -163: Please be specific on the exact times when samples were collected. What time in the morning? How many samples were collected within 48 hours and how many within 72 hours. These are important because there are established variations in the cytokine concentrations with time.

Line 156: This should be under the results section

Discussion

Line 232-233: Please modify

Line 281-286: IL-6 is proinflammatory in the acute phase and involved in immune regulation of the inflammatory response. Its secretion is augmented by TNF-α and IL-1 β so it is not surprising that both are elevated although TNF-α is not. Considering that these are elevated in pre-eclampsia and sickle cell disease in pregnancy, it seems that the role of IL-1 β in augment IL-6 secretion in pregnancy is highly significant compared to TNF-α. Incidentally the risk group for preeclampsia is maternal age >35 years. This is quite interesting in understanding the inflammatory environment in pregnancy.

Conclusion:

The data is relevant and the article is publishable after the corrections are made.
