# Supplementary material for: Profile of pro-inflammatory cytokines in colostrum of nursing mothers at the extremes of reproductive age
Source: PLoS One. 2020 Jun 16;15(6):e0231882. doi: 10.1371/journal.pone.0231882 (PMC7297348; doi:10.1371/journal.pone.0231882)
Supplement: Attachment — Submitted filename: Response to Reviewers_2.docx [file pone.0231882.s003.docx]

March 30 2020

**Review:** Manuscript number **PONE-D-19-35580**

Editorial Board, PLOS ONE

Thank you for the careful review of our work and for providing us the opportunity to submit a revised version of the manuscript that addresses the points below.

**All answers are detailed below each reviewers’ comments, as well highlighted in the revised version of the manuscript.**

**REVIEW COMMENTS TO THE AUTHOR**

**Reviewer #1**: Judged by the responses from the authors, most of the problems are resolved in the revised version. However, there remains some points for correction.

**Answer:** We appreciate your careful review, and the suggested changes were performed, as highlighted in blue in the revised version of the manuscript in the respective lines named below.

1. Lines 41-42: “immunoenzymatic assay (ELISA)” should be changed to “enzyme-linked immunosorbent assay (ELISA)” (Line 41-42).

2. Line 149: “72h” should be changed to “72 h” (Line 149).

4. Line 154: “2mL” should be changed to “2 mL” (Line 154).

5. Line 155: “160g” should be changed to “160 g” (Line 155).

6. Line 189: “IL-8 e TNF-α” should be changed to “IL-8, and TNF-α” (Line 159).

7. Lines 173-174: “Graph Pad Prism software, version 6.0” should be changed “Graph Pad Prism software, version 6.0 (San Diego, CA)” (Line 173-174).

**Reviewer #2:** The question raised previously have been well addressed. There are a few typographic errors as indicated below:

**Answer:** We appreciate your careful review, and the suggested changes were performed, as highlighted in blue in the revised version of the manuscript in the respective lines named below.

1. Abstract: Line 41: attended at the.....should be replaced with: who attended the maternity—The study’s location had been suppressed from the abstract in the first revision, we include the suggestion grammar in the Methods section (Line 124)

2. line 139:... should be punctuated as: advanced maternal age. This...(Line 140)

3. line 153: should be punctuated as: give birth. (included in the study group : should be deleted.)

We thank the reviewer and clarify that paragraph was rephrased in the revised version (Lines 139-141)

Hope these changes are satisfactory and will consider our paper for publication in PLoS ONE.

Sincerely,

Morceli, G
